# Impact of tumor necrosis factor antagonist combination and anti-integrin therapies on body mass index in inflammatory bowel disease: A cross-sectional study

**DOI:** 10.3389/fmed.2022.1045661

**Published:** 2023-01-06

**Authors:** Mohammad Shehab, Ali Alali, Ahmed Al-Hindawi, Abdulwahab Alsayegh, Usama Aldallal, Israa Abdullah, Abbas Albaghli, Fatema Alrashed, Ahmad Alfadhli, Talat Bessissow

**Affiliations:** ^1^Department of Internal Medicine, Faculty of Medicine, Mubarak Al-Kabeer Hospital, Kuwait University, Jabriya, Kuwait; ^2^School of Medicine, Royal College of Surgeons in Ireland, Medical University of Bahrain, Al Muharraq, Bahrain; ^3^Department of Pharmacy, Kuwait Hospital, Sabah Al-Salem, Kuwait; ^4^Division of Gastroenterology, Montreal General Hospital, McGill University Health Center, Montreal, QC, Canada; ^5^Department of Pharmacy Practice, Faculty of Pharmacy, Health Sciences Center (HSC), Kuwait University, Jabriya, Kuwait

**Keywords:** IBD, obesity, medications, biologics, BMI

## Abstract

**Background:**

The impact of biologic therapies on body mass index (BMI) in patients with inflammatory bowel disease (IBD) is unclear. This study investigates any associations between BMI, type of IBD, and the type of medications taken among patients with IBD with varying weight categories.

**Methods:**

A cross sectional study was performed in an IBD tertiary care center. Data was obtained from patients with IBD attending outpatient clinics from January 1st, 2021 until November 1st, 2021. Adult patients, older than 18 years, with a diagnosis of Crohn’s disease (CD) or ulcerative colitis (UC) were recruited. The primary outcome was the association between BMI and medication used in IBD. The secondary outcome was the association between BMI and disease type and location in patients with IBD.

**Results:**

The study included a total of 528 patients of which, 66.5% have CD. Patients with normal weight comprises 55.9% of the participants, while those who are underweight, overweight or obese are 3.4, 28.2, and 12.5%, respectively. None of the underweight patients had UC. Among the normal weight, overweight and obese BMI categories, 34.6% vs. 36.2% vs. 31.8% had UC, respectively. Patients who are on tumor necrosis factor inhibitors (anti-TNF) with an immunomodulator (anti-TNF combination), are more likely to be overweight or obese than patients who are not on anti-TNF combination (OR 2.86, 95% CI 1.739–4.711, *p* < 0.001). Patients on vedolizumab are twice as likely to be overweight or obese than patients not on vedolizumab (OR 2.23, 95% CI 1.086–4.584, *p* < 0.05). Patients with ileocolonic CD are more likely to be overweight or obese compared to other subtypes of CD (OR 1.78, 95% CI 1.14–2.77, *p* = 0.01).

**Conclusion:**

Many patients with IBD are either obese or overweight. Patients with IBD who are on anti-TNF combination therapy or vedolizumab monotherapy are more likely to be obese and overweight. In addition, patients will ileocolonic CD are more likely to be obese or overweight.

## 1. Introduction

Inflammatory bowel disease (IBD) is a chronic immune-mediated inflammatory disorder with two common types: ulcerative colitis (UC) and Crohn’s disease (CD). It is thought to be caused by a complex combination of genetic and environmental factors. IBD is commonly treated with immunosuppressive and biologic agents such as corticosteroids, tumor necrosis factor inhibitors (anti-TNF), thiopurines, anti-integrins, IL12/IL23 inhibitors, and janus kinase inhibitors (1). Given that many symptoms such as changes in bowel movement or bloody stools are often associated with IBD, a commonly feared consequence is malnourishment and an increased likelihood of weight loss or being underweight. However, reported malnutrition rates among IBD patients internationally can vary considerably, with a prevalence anywhere between 5.7 and 82.8% (2). While IBD can often be linked to underweight patients, IBD is increasingly occurring in obese patients (3). In the general IBD population, 20–30% of both adult and pediatric patients are overweight or obese as per standard definitions. Moreover, records of the weight distribution among IBD patients suggest a clear trend of increased obesity impacting patients with CD rather than UC (4).

Obesity is often defined as a body mass index (BMI) equal to or more than 30 kg/m^2^. The cause of obesity is often heterogeneous and multifactorial, involving metabolic, social, cultural, behavioral, and genetic factors (5, 6). Over the past few decades, the prevalence of obesity has been increasing and recognized as a public health concern. Indeed, roughly one-third of Americans are obese and in the last 10 years, the prevalence of obesity in children in Europe has drastically increased from 10 to 40% (6). In Saudi Arabia, 34.8% of the adult population is considered obese, and in Kuwait, 40.9% of children between 6 and 8 years of age are considered overweight or obese (7). As such, health concerns are emerging given obesity is continuing to rise. Diabetes mellitus, hypertension, cardiovascular disease, and kidney injury are just some of the associated adverse health conditions that obesity can result in (World Health Organization) 8. Recent data suggests that the proportion of IBD patients who are obese mirrors the proportion in the general population, which is between 15 and 40% (9).

Over time, IBD and obesity have increased in parallel among the same population, suggesting a possible association (3). Obesity generates a proinflammatory state through the secretion of inflammatory mediators including tumor necrosis factor (TNF)-a and C-reactive protein (CRP) (10). Therefore, questions of possible association between anti-TNFs and other biologics with increased BMI have been raised (11). Thus, this study aims to investigate any associations between BMI and the different group of medications taken among patients with IBD with varying weight categories.

## 2. Materials and methods

A cross-sectional study was performed at an IBD tertiary care center, Mubarak Alkabeer University Hospital in Kuwait. Data from patients with IBD attending outpatient clinics from January 1st 2021 until Nov 1st 2021, was obtained. Adult patients older than 18 years with a diagnosis of CD or UC were included. Patient demographics and detailed patient characteristics were recorded according to Montreal’s classification. In addition, records of lifestyle factors such as smoking, BMI, and other comorbid conditions were obtained. Data were taken from patients’ electronic health records and patients charts. BMI categories were selected based on World Health Organization international classifications (BMI < 25 kg/m^2^, normal; 25–29.9 kg/m^2^, overweight; ≥ 30 kg/m^2^, obese) (12). To ensure reliability of the results, BMI of patients was recorded on two separate visits, at least 8 weeks apart, to confirm patients remain in the same BMI category. Medication history of specific IBD medications such as mesalamine, thiopurines, methotrexate, and biologics was recorded.

Stool fecal calprotectin > 250 μg/mg, serum CRP > 10 mg/L or serum albumin < 40 g/L, for the purpose of this study, were accounted for in our analysis and were called “inflammation.” In addition, clinical scores, partial Mayo score for UC and Harvey-Bradshaw Index (HBI) score for CD, were recorded. For the purpose of this study, patients on infliximab, adalimumab, vedolizumab or ustekinumab for at least 6 weeks were considered to be on biologics. Patients taking infliximab or adalimumab concomitantly with an immumodulator (methotrexate or azathioprine) were considered to be on anti-TNF combination therapy. Moreover, patients taking mesalamine, azathioprine, methotrexate without any biologic medication for at least 6 weeks were considered to be on non-biologics.

Our exclusion criteria included: patients who have other chronic autoimmune diseases, such as rheumatological disease, patients who were chronic medications that can cause weight gain were excluded, such as neuropsychiatric medications, patients who were on chronic steroid use, more than 8 weeks, patients with disease that can cause weight gain were excluded such as diabetes, congestive heart failure, endocrine diseases, renal disease, and liver cirrhosis. In addition, basic laboratory tests were checked (full blood count, renal function tests, liver function tests, HbA1c) to objectively screen for underlying diseases and if found abnormal patients were excluded. Finally, patients whose BMI changed categories within study period were excluded.

This study was performed and reported in accordance with Strengthening the Reporting of Observational Studies in Epidemiology (STROBE) guidelines (Supplementary Table 1) (13). The international classification of diseases (ICD-10 version: 2016) was used to make diagnosis of IBD. Patients were considered to have IBD when they had ICD-10 K50, K50.1, K50.8, K50.9 corresponding to CD and ICD-10 K51, K51.0, K51.2, K51.3, K51.5, K51.8, K51.9 corresponding to UC (14).

The study protocol was reviewed and approved by the standing committee for coordination of health and medical research at the ministry of health of Kuwait (reference: 3613, protocol number 3679/2021).

### 2.1. Outcome measures

The primary outcome was to investigate the association between BMI and IBD medications use in patients with IBD. The secondary outcome was to investigate the association between IBD type or subtype, as per Montreal classification (15), in patients with IBD.

### 2.2. Statistical analysis

Descriptive statistics were carried out and reported as mean ± standard deviation or percentages. Between-group comparisons were carried out between different weight categories (based on BMI) for all variables using the chi-square test and *t*-test, where appropriate. Multivariable logistic regression analyses were performed to assess the association between different weight groups and medical therapy for IBD and to assess the relationship between IBD subtypes and being overweight/obese. The results of the regression model were presented as odds ratios (OR) with a null value of “1.” Statistical significance was set to a *p*-value < 0.05 and confidence interval (CI) of 95% for all associations. All associations were adjusted for the effects of sex, inflammation, and age. All analyses were performed using Stata software version 15.1 (Stata Corp LP, College Station, TX, USA).

To account for possible confounders, *t*-test was performed to examine the possible association between the use of biologic medications and disease severity, expressed as partial Mayo score for UC and HBI score for CD. Moreover, other potential confounders, such as age, sex and inflammation were controlled for in all the performed analyses. Inflammation was expressed as CRP, albumin and stool fecal calprotectin levels.

## 3. Results

### 3.1. Demographics

Patients baseline characteristics are shown in Table 1. A total of 528 patients are included in the study, of which 304 patients (57.6%) are males and 351 (66.5%) had CD. Mean age is 34.3 (±12.8) years, and most are non-smokers [*n* = 440 (83.3%)]. 324 patients (61.4%) are on biologics, of which 188 (58%) are on infliximab, 52 (16.1%) on adalimumab, 46 (14.2%) on vedolizumab and 38 (11.7%) on ustekinumab. Most of the patients included, 295 (55.9%), have a normal BMI, while those who are overweight or obese are 149 (28.2%) and 66 (12.5%) patients, respectively. Interestingly, 215 (40.7%) patients were either overweight or obese. Only 18 (3.4%) of the participants are underweight (Table 1). Participants have similar baseline characteristics in most aspects.

**TABLE 1:** Baseline characteristics of the entire cohort.

Variables	Total, *N* = 528
Age (years), mean (SD)	34.3 (12.8)
**Sex, *n* (%)**	
Male	304 (57.6)
Female	224 (42.4)
**Smoking, *n* (%)**	
Yes	88 (16.7)
No	440 (83.3)
**IBD type, *n* (%)**	
Ulcerative colitis	177 (33.5)
Crohn’s disease	351 (66.5)
**Ethnicity, *n* (%)**	
Middle-Eastern	498 (94.3)
Others	30 (5.7)
Body mass index (BMI)	
Mean (SD)	25.0 (4.9)
**Weight category, *n* (%)**	
Underweight (BMI < 18.5)	18 (3.4)
Normal (BMI = 18.5–24.9)	295 (55.9)
Overweight (BMI 25–29.9)	149 (28.2)
Obese (>30)	66 (12.5)
**Ulcerative colitis (UC) type, *n* (%)**	
E1: proctitis	53 (29.9)
E2: left-sided	49 (27.7)
E3: extensive	75 (42.4)
Missing	0 (0)
**Crohn’s disease (CD) location, *n* (%)**	
L1: ileal	185 (52.7)
L2: colonic	58 (16.5)
L3: ileocolonic	105 (29.9)
L4: upper gastrointestinal	3 (0.9)
Missing	0 (0.0)
**Crohn’s disease (CD) phenotype, *n* (%)**	
B1: non-stricturing, non-penetrating	176 (50.1)
B2: stricturing	52 (14.8)
B3: penetrating	34 (9.7)
Missing	98 (27.9)
**Crohn’s disease (CD) perianal disease, *n* (%)**	
Yes	74 (21.1)
No	277 (78.9)
**Steroid use, *n* (%)**	
Yes	36 (6.8)
No	492 (93.2)
**Medical therapy, *n* (%)**	
Biologic	324 (61.4)
Non-biologic	204 (38.6)
Non-biologic therapy, *n* (%) (*n* = 204)*	
Mesalamine	108 (52.9)
Azathioprine	105 (51.5)
Methotrexate	3 (1.5)
**Biologics, *n* (%) (*n* = 324)**	
Adalimumab	52 (16.1)
Infliximab	188 (58.0)
Ustekinumab	38 (11.7)
Vedolizumab	46 (14.2)
**Biologics use duration, *n* (%)**	
<6 months	32 (9.9)
6–12 months	36 (11.1)
1–5 years	198 (61.1)
>5 years	58 (17.9)

*Some patients taking ≥ 1 medication.

BMI, body mass index; CD, Crohn’s disease; GI, gastrointestinal; IBD, inflammatory bowel disease; SD, standard deviation; UC, ulcerative colitis; N/A, non-applicable.

No difference was found in the baseline inflammatory markers and clinical scores between patients who are on biologics and those who are not on biologics (Table 2). Patient’s demographics based on the BMI categories are presented in Supplementary Table 2. Patients who were overweight were older than patients in the other groups with mean age 37.7 (±13.9) years compared to 33.4 (±12.7) years and 33.7 (±9.1) years for patients with normal weight and obese, respectively, *P* < 0.05. Among underweight patients, 12 (66.7%) were males. Males comprised 161 patients (54.6%) of the normal weight, 95 (63.8%) of the overweight and 36 (54.5%) of the obese BMI categories (*P* = 0.2). Moreover, the proportions of smokers were significantly different across the BMI categories. There were 10 (55.6%) smokers with underweight, 47 (15.9%) with normal weight, 24 (16.1%) with overweight, and 59 (89.4%) obese, *P* < 0.05 (Supplementary Table 2).

**TABLE 2:** *T*-test for the mean baseline levels of inflammatory markers and clinical scores between patients with Crohn’s disease or ulcerative colitis who are on biologics vs. non-biologics.

	Biologics	Non-biologics	*P*-value
**Crohn’s disease, *n* = 351**			
Number of patients (%)	240 (68.38%)	111 (31.62%)	
Fecal calprotectin	141.43	132.05	0.499
C-reactive protein	8.03	7.19	0.078
Albumin	38.80	38.67	0.359
Harvey-Bradshaw Index (HBI) score	2.50	2.24	0.346
**Ulcerative colitis, n = 177**			
Number of patients (%)	84 (47.46%)	93 (52.54%)	
Fecal calprotectin	130.40	137.66	0.620
C-reactive protein	7.81	8.42	0.222
Albumin	38.99	38.99	0.829
Partial Mayo score	1.45	1.27	0.242

*Values are presented as mean—All associations were adjusted for the effects of sex, inflammation, and age.

### 3.2. Primary outcomes

All 18 patients (100%) in the underweight category were on biologics, in contrast to 160 (54.2%), 98 (65.8%), and 48 (72.7%), in the normal weight, overweight, and obese BMI categories respectively (*P* < 0.05). On the other hand, none of the underweight patients were on a non-biologic medication. On univariate analysis there was no significant association between the use of any single immunomodulator or biologics and BMI. However, the use of anti-TNF combination was significantly different among the BMI categories. There were 8 (44.4%) patients on anti-TNF combination in the underweight category compared to 46 (38.3%), 52 (74.3%), and 12 (37.5%) in the normal weight, overweight, and obese BMI categories respectively (*P* < 0.05). Conversely, no significant difference was found between steroid use across the BMI categories. Two patients (11.1%) who were on steroids were underweight as opposed to 16 (5.4%) patients, 14 (9.4%) patients and 4 (6.1%) patients in the normal weight, overweight, obese categories respectively (*P* = 0.1, Supplementary Table 2).

On multivariant analysis, the associations between the use of biologic medications and being an overweight or obese compared to having normal weight are shown in Table 3. Patients who are on anti-TNF combination are more likely to be overweight or obese than patients who are not on anti-TNF combination (OR 2.86, 95% CI 1.739–4.711, *p* < 0.001). Similarly, patients on vedolizumab are twice as likely to be overweight or obese than patients not on vedolizumab (OR 2.23, 95% CI 1.086–4.584, *p* < 0.05). No significant association is found between the use of ustekinumab, infliximab, or adalimumab alone and being overweight or obese (Table 3).

**TABLE 3:** Association between biologic medications use and being overweight or obese compared to having normal weight in patients with inflammatory bowel disease (IBD).

Medications	Odds ratio*	*P*-value	95% CI	
			Lower	Upper
Anti-TNF combination	2.862	0.000	1.739	4.711
Infliximab	1.050	0.862	0.608	1.811
Adalimumab	0.858	0.710	0.383	1.924
Vedolizumab	2.231	0.029	1.086	4.584
Ustekinumab	1.717	0.147	0.827	3.568

*Adjusted for age, sex and inflammation. CI, confidence interval.

### 3.3. Secondary outcomes

None of the underweight patients have UC as opposed to 18 (100%) patients with CD. Among patients with IBD, UC comprises 102 (34.6%) of the normal weight group, 54 (36.2%) in the overweight and 21 (31.8%) in the obese BMI groups compared to CD (*P* < 0.02). Moreover, there is an in-between groups statistical difference between UC subtypes across the BMI categories. The normal weight group consists of 30 (29.4%) patients with proctitis, 26 (25.5%) with left-sided disease, and 46 (45.1%) with extensive UC. Additionally, overweight group includes 23 (42.6%) patients with proctitis, 16 (29.6%) left-sided disease, and 15 (27.8%) with extensive UC. In the obese group, none of the UC patients have proctitis as opposed to 7 (33.3%) with left-sided disease, and 14 (66.7%) with extensive UC (*p* < 0.05). No in-between groups difference was found across CD subtypes (location, phenotype, or perianal disease) and BMI categories (*p* < 0.2, *p* < 0.3, and *p* < 0.3, respectively, Supplementary Table 2).

Associations between IBD subtypes and having overweight or obesity is shown in Table 4. In the multivariate analyses, no association was found between having UC or CD and being overweight or obese (OR 1.027, 95% CI 0.702–1.502, *p* = 0.89). Similarly, no significant association was observed between the different subtypes of UC (proctitis, left sided UC, and extensive UC) and having overweight or obesity (OR 1.093, 95% CI 0.618–1.93, *p* = 0.76, OR 1.205, 95% CI 0.656–2.212, *p* = 0.548, OR 0.844, 95% CI 0.505–1.411, *p* = 0.617, respectively). Moreover, no significant relationship was found between any of the CD phenotypes and overweight or obesity (non-stricturing: OR 0.746, 95% CI 0.506–1.101, *p* = 0.140, stricturing: OR 0.82, 95% CI 0.445–1.511, *p* = 0.524, and penetrating OR 1.784, 95% CI 0.869–3.664, *p* = 0.115).

**TABLE 4:** Association between the type of inflammatory bowel disease (IBD) and being overweight or obese compared to having underweight or normal weight (total sample = 528).

	Odds ratio (OR)*	*P*-value	95% CI	
			Lower	Upper
**Type of inflammatory bowel disease (IBD)**				
Ulcerative colitis (UC)	1.027	0.89	0.702	1.502
Crohn’s disease (CD)**				
**Ulcerative colitis (UC) type**				
E1: proctitis	1.093	0.760	0.618	1.930
E2: left-sided	1.205	0.548	0.656	2.212
E3: extensive	0.844	0.517	0.505	1.411
**Crohn’s disease (CD) phenotype**				
B1: non-stricturing, non-penetrating	0.746	0.140	0.506	1.101
B2: stricturing	0.820	0.524	0.445	1.511
B3: penetrating	1.784	0.115	0.869	3.664
**Crohn’s disease (CD) location**				
L1: ileal	0.698	0.072	0.472	1.033
L2: colonic	1.190	0.545	0.677	2.091
L3: ileocolonic	1.782	0.011	1.145	2.776
L4: upper GI	<0.00	0.99	<0.00	<0.00
Perianal disease	1.268	0.357	0.765	2.104

*Logistic regression model is run separately for each subtype and adjusted for age, sex and inflammation, CI, confidence interval.

**Reference group to ulcerative colitis (UC).

All associations were adjusted for the effects of sex, inflammation, and age.

With the exception of ileocolonic Crohn’s disease, CD subtypes based on disease location, including ileal, colonic, upper GI and perianal disease, were not associated with having overweight or obesity (OR 0.698, 95% CI 0.472–1.033, *p* = 0.072, OR 1.19, 95% CI 0.677–2.091, *p* = 0.545, OR < 0.00, 95% CI < 0.00–0.00, *p* = 0.99, OR 1.268, 95% CI 0.765–2.104, *p* = 0.357, respectively). However, patients with ileocolonic CD were almost twice as likely to be overweight or obese compared to patients with other CD subtypes (OR 1.782, 95% CI 1.145–2.776, *p* = 0.011, Table 4).

## 4. Discussion

The current study aimed to explore the potential associations between BMI and different IBD medications in patients with IBD. We found that patients treated with anti-TNF combination therapy or vedolizumab are more likely to be overweight or obese. No significant association is found between the use of ustekinumab, infliximab, or adalimumab alone and being overweight or obese.

The evidence on the relationship of obesity and anti-TNF combination therapy is limited, however, a systematic review and meta-analysis explored differences between pre and post- anti-TNF therapy on patients’ weight. The meta-analysis revealed that patients’ weight was significantly higher after anti-TNF initiation, with infliximab having the most significant effect on patients’ BMI (16). Another systematic review and meta-analysis explored changes in body weight and BMI in patients with psoriasis after receiving various biologics (17). Similarly, authors found that treatment with anti-TNFs appears to be associated with an increase in body weight and BMI, and treatment with anti-IL-12/23 and anti-IL-17 biologics do not. A possible explanation of anti-TNF effect on BMI can be attributed to the fact that TNF-α inhibition might affect both the central as well as the peripheral mechanisms regulating body weight (18).

Studies focusing on vedolizumab in the context of overweight or obese patients with IBD are particularly scarce. In fact, to our knowledge, the present paper is the first to report a significant association between vedolizumab use and overweight/obesity. A possible reason is that obese patients are more likely to get prescribed vedolizumab because of its safety profile and a non-weight-based fixed dose (19).

Within our studied population, approximately 40.7% were overweight or obese, with only 3.4% of the population classified as underweight. This is in sharp contrast with older, perhaps now historical, studies. For instance, a 2002 study involving 2065 French CD patients demonstrated an obesity prevalence of only 3% (20). Furthermore, an older paper studying 70 Scottish patients with IBD reported a significant difference in weight between CD and UC patients (men: 66.8 vs. 78.4 kg; women: 51.2 vs. 63.0 kg) (21). Yet, more recent studies highlight the ever-increasing prevalence of obesity among patients with IBD (22). In a study conducted by Moran et al., 10,282 patients from forty randomized controlled trials conducted from 1991 to 2008 were included in a time-trend analysis. The group highlighted a significant increase in weight and BMI over time; clinical disease activity was simultaneously increased (23). Contrary to previously held beliefs, obesity rates among adult patients with IBD are estimated to be between 15 and 40%, a figure that is similar to our findings (9).

In our study, multivariate analyses showed no association between having UC or CD and being overweight or obese. In a large prospective U.S. study, a group of 382 patients with IBD was followed for a total of over 2 million person-years. Obesity was associated with the development of CD, with hazard ratios of 2.33 at age 18. Weight gain was also associated with an increased risk of CD; however, such results were not observed in UC (24). Likewise, a systematic review incorporating 23,371 cases of IBD concluded that obesity increases IBD risk (HR 1.20; 1.08–1.34) (25). A gradual increase in BMI has been documented to increase the risks of CD. In a pooled analysis study conducted by Chan et al., each 5-kg/m^2^ increase in BMI increases the risk of CD by 16%, where a BMI ≥ 30 kg/m^2^ was associated with a 34% increased risk of developing CD (9) (3). The exact underlying pathophysiology of how obesity perpetuates IBD development is not fully understood but is thought to involve multiple processes: [1] increased systemic levels of chemokines, cytokines, and adipokines; [2] proinflammatory gene expression profile; [3] increased numbers of proinflammatory subtypes of immune cells in hypertrophic fat tissue; and [4] reduction in gut bacterial diversity and dysbiosis (26).

Another important finding of this study is that patients with ileocolonic CD are almost twice as likely to be overweight or obese compared to patients with other CD subtypes. While a previous study observed no differences with regards to disease subtypes (B1–B3 in CD) and extent (E1–E3 in UC) between obese and normal weight patients, the same study found that obese CD, but not UC patients, showed increased disease activity scores compared to normal weight patients (27). Despite impacting the disease’s pathophysiology and natural course, the current evidence is inconclusive on whether obesity worsens disease severity or complication rates (9). Studies have shown a poorer quality of life scores and higher levels of inflammatory markers in obese patients; however, no significant variation in emergency admissions, hospitalization, and IBD-related surgeries was identified between obese and non-obese patients (28). In fact, a study by Flores et al. reported a potential protective effect of obesity on IBD complications where hospitalizations and IBD-related surgery were lower in the obese group compared to the normal or underweight group (29).

The current study has several clinical implications. Given the alterations in pharmacokinetics and comorbidities that obesity introduces, IBD treatment plans and their efficacies may be influenced. In our study population, 67.9 and 62.7% of overweight and obese patients are on biologics and anti-TNF combination therapy, respectively. Conversely, only 54.2 and 38.3% of normal-weight patients are on biologics and anti-TNF combination therapy. Considering the inflammatory nature of obesity, where higher TNFα levels are secreted by adipose tissue, the efficacy of anti-TNF agents may be negatively affected. Unfortunately, the evidence on the effect of obesity on IBD anti-TNF therapy is currently conflictive and non-conclusive. In a retrospective study involving 124 patients with IBD, an approximately threefold increase in loss of response to infliximab was seen in obese CD patients compared to non-obese CD patients (30). In this study, an increase of 1 kg/m^2^ in BMI corresponded to a 6% higher chance of an acute CD flare. A meta-analysis performed by Singh et al. showcased that obesity was associated with a higher rate of anti-TNF treatment failure within IBD patients (31). Similar to anti-TNFs, high BMI may have an adverse effect on vedolizumab efficacy. Indeed, higher vedolizumab trough levels have been associated with better clinical outcomes (32, 33), and an inverse relationship between vedolizumab trough levels and weight has been reported (34). This paradigm is partially supported by a recent 2022 single-center retrospective study, where obesity was associated with lower rates of vedolizumab discontinuation, CRP normalization and higher rate of endoscopic remission (19).

There are several strengths to our study. It is a well-designed cross-sectional study in which we controlled for several important confounders, such as inflammation and clinical scores. It also the first study in the region to describe such findings. In addition, it is the first study, to our knowledge, to describe an association between vedolizumab and obesity. However, our study has some limitations. Given the nature of observational studies, there could be some potential confounders that were not accounted for. In this study, data were collected from only a single center. Furthermore, our observed associations do not demonstrate causality and may be related to other confounding factors such as lifestyle measures and change in BMI over long period of time, which we were unable to adjust for. Also, previous surgeries were not accounted for which may be associated with change in BMI.

## 5. Conclusion

In conclusion, many patients with inflammatory bowel disease (IBD) are either obese or overweight. Patients with IBD who are on anti-TNF combination therapy or vedolizumab monotherapy are more likely to be obese and overweight. There was no association between other medical therapies for IBD and obesity or overweight. In addition, patients with ileocolonic Crohn’s disease are more likely to be obese or overweight. Physicians should be aware that obesity is rising in patients with IBD which may affect their treatment efficacy and increase risk of other co-morbidities.

## Data availability statement

The raw data supporting the conclusions of this article will be made available by the authors, without undue reservation.

## Ethics statement

The study protocol was reviewed and approved by the Standing Committee for Coordination of Health and Medical Research at the Ministry of Health of Kuwait (reference: 3613, protocol number: 3679/2021). Written informed consent for participation was not required for this study in accordance with the national legislation and the institutional requirements.

## Author contributions

MS: conceptualization, methodology, validation, investigation, resources, data curation, and writing—review and editing. AAla and IA: software and formal analysis. AA-H, AAls, and UA: writing—original draft preparation. AAlb: data curation. FA: data curation and writing—review and editing. AAlf: supervision. TB: supervision and writing—review and editing. All authors contributed to the article and approved the submitted version.
